# When Stigma Differs: A Cross‐Sectional Study of Divergent Associations of Internalized and Enacted Stigma on Human Immunodeficiency Virus Disclosure in Zambia

**DOI:** 10.1002/hsr2.72800

**Published:** 2026-07-09

**Authors:** Joshua Okyere, Mutinta Monze

**Affiliations:** ^1^ School of Human and Health Sciences University of Huddersfield, Queensgate Huddersfield England UK; ^2^ College of Health Sciences University of Ghana Accra Ghana; ^3^ Department of Obstetrics and Gynaecology Choma General Hospital Choma Zambia

**Keywords:** disclosure, HIV, stigma, Zambia

## Abstract

**Background:**

In Zambia, HIV is a serious public health concern that predominantly affects women of reproductive age. Disclosure of HIV status remains central to treatment adherence, partner notification, and prevention efforts. Yet stigma continues to constrain disclosure. However, the question remains that: are all stigma the same? How different does internalized and enacted stigma affect HIV status disclosure? To answer these questions, the study examined the association between internalized, enacted stigma, and HIV status disclosure among women in Zambia.

**Methods:**

This study analyzed the 2024 demographic and health survey data of 1037 women living with HIV in Zambia. The outcome variable was HIV status disclosure, with the key exposure variables being internalized and enacted stigma. A multivariable logistic regression was performed to assess the association. Statistical significance was set at *p* < 0.05.

**Results:**

Overall, 89.7% (95% CI: 87.5–91.5) of women reported having disclosed their HIV status. About 26.1% and 21.8% experienced internalized and enacted stigma, respectively. The findings also revealed that women living with HIV mostly disclosed their status to family members (48.2%), followed by their intimate partners (29.7%). Women who experienced internalized stigma had significantly lower odds of HIV disclosure [AOR = 0.52; 95% CI: 0.33–0.84]. However, enacted stigma showed no statistical significance.

**Conclusion:**

HIV disclosure among women in Zambia is high but remains patterned by internalized stigma and HIV transmission knowledge. Integrating stigma‐informed counseling with targeted health education may enhance disclosure and support broader HIV prevention and care outcomes.

**Abbreviations**
AOR: adjusted odds ratio
ART: antiretroviral therapy
CI: confidence interval
COR: Crude odds ratio
HIV: human immunodeficiency virus
PLHIV: persons living with HIV
SSA: sub‐Saharan Africa
WHO: World Health Organization
ZDHS: Zambia Demographic and Health Survey

## 1 Background

Human immunodeficiency virus (HIV), a viral infectious disease, has been one of the world's top health priorities for years. As of the end of 2024, there were approximately 40.8 million people living with HIV (PLHIV); of this number, 65% were located in Africa [1]. The extant evidence indicates that Southern sub‐Saharan Africa (SSA) has the highest age‐standardized incidence rate of HIV compared to any other part of the world [2]. Zambia happens to be located in this region, and the situation is not different. One study reports a high recent HIV infection prevalence of 34%, with women bearing the highest burden [3]. Another study based on the Zambia population‐based HIV impact assessment reports an overall HIV prevalence of 11% [4].

Zambia's HIV response, like other countries, is shaped by the Joint United Nations Programme on HIV/AIDS (UNAIDS) 95‐95‐95 targets, which emphasizes that at least 95% of PLHIV will know their status; 95% will be on treatment; and 95% achieve suppressed viral load by the year 2025 [5]. However, as at the end of 2024, only 77% of PLHIV worldwide were on treatment—a figure that is below the 95% target [1]. Zambia has generally performed well in the area of viral load suppression (96.6%) and treatment (98.4%); however, the proportion that knows its HIV status is still far below the 95% mark (90.1%) [4, 6]. Moreover, there are significant disparities in these indicators as viral suppression in regions such as Luapula (94.1%), Muchinga (93.6%), Northern (92.6%), and North‐Western (90.4%) falls below the 95% threshold [6]. This is where HIV status disclosure could play a role.

Studies have shown that HIV status disclosure plays a critical role in facilitating timely treatment initiation, adherence to antiretroviral therapy (ART), partner notification, and psychosocial support [7]. All of these are necessary for the attainment of a 95% treatment adherence and 95% viral load suppression. Zurashvili et al. [7] argue that HIV status disclosure fosters empowerment, which is necessary for PLHIV to seek treatment and reduced HIV transmission. Beyond treatment adherence, HIV status disclosure may foster emotional support, reduce psychological distress, facilitate safer sexual practices, improve partner communication, strengthen social connectedness, and encourage uptake of prevention services among exposed partners and family members [7, 8].

Despite the positive impact of HIV status disclosure on treatment seeking, treatment adherence, and reduced HIV transmission [7], it suffers several challenges. One of such challenges is stigma. Stigma operates through multiple pathways, commonly conceptualized as internalized stigma, which reflects negative self‐perceptions related to HIV status [9], and enacted stigma, which refers to experiences of discrimination or mistreatment by others [10]. While the Global AIDS Strategy 2021–2026 calls for HIV stigma to be reduced to < 10% [11], the available evidence indicates that in Zambia, about 23.6% and 4.3% of PLHIV experienced stigma in their community and at healthcare facilities, respectively [12]. Moreover, the evidence from Zambia also shows that women often experienced HIV stigma in the community and healthcare setting, compared to their male counterparts [13].

Both forms of stigma have been associated with reduced care engagement, poorer mental health outcomes, and diminished social support. For example, stigma discourages individuals from attending HIV clinics regularly, collecting antiretroviral medications, participating in support groups, or openly discussing their health concerns with healthcare providers due to fear of judgment or social exclusion [10, 12]. Felker‐Kantor et al. [14] revealed that PLHIV who experienced internalized stigma were 67%, 91%, and 45% more likely to suffer depressive symptoms, anxiety symptoms, and hazardous drinking, respectively. Hong et al. [15] also revealed that PLHIV who experienced any form of stigma experienced significantly lower quality of life and life satisfaction. This makes HIV stigma an important public health concern. While the extant literature has explored the prevalence of HIV stigma [12, 13], and its effect on mental health outcomes [14] and quality of life [15], a fundamental question remains answered. Is all stigma the same? How different does internalized and enacted stigma affect HIV status disclosure? To answer these questions, the study examined the association between internalized, enacted stigma, and HIV status disclosure among women in Zambia. The findings of this study are essential for designing effective, gender‐responsive interventions.

## 2 Methods

### 2.1 Study Design

For this study, we performed a secondary data analysis based on the 2024 Zambia Demographic and Health Survey (ZDHS). Zambia is a lower‐middle‐income country in Southern Africa with one of the highest HIV burdens globally [6]. Women continue to experience disproportionate HIV‐related vulnerabilities due to gender inequalities, socioeconomic challenges, and persistent HIV‐related stigma. The country's high HIV prevalence and ongoing national HIV response initiatives make Zambia an important setting for examining HIV disclosure practices and stigma experiences [3, 4, 6].

The ZDHS is a nationally representative, cross‐sectional household survey implemented using a stratified two‐stage cluster sampling design [16]. Enumeration areas were selected at the first stage, followed by systematic sampling of households within each cluster. First, 545 clusters were selected from 20 urban/rural provincial strata using probability proportional to size. After a full listing of an average 111 households per cluster, 25 households were chosen from each via equal probability systematic sampling, totaling 13,625 households [17]. The survey collected detailed information on sociodemographic characteristics, HIV knowledge, stigma‐related experiences, and HIV‐related behaviors among women aged 15 to 49 years [16]. To maintain confidentiality, interviews were conducted in private settings, and participants’ identifying information was anonymized within the DHS dataset. These safeguards were particularly important given the sensitive nature of HIV disclosure and stigma‐related questions [16]. Our analytic sample comprised 1037 women who self‐reported living with HIV and had complete information on the outcome variable.

### 2.2 Study Variables

#### 2.2.1 Outcome

HIV status disclosure was the outcome variable. This was defined as whether respondents self‐reported having disclosed their HIV status to others (i.e., family member, partner, friends, healthcare professional, religious leader, or others). This variable was coded dichotomously. Responding “yes” meant that the PLHIV had disclosed their HIV status.

#### 2.2.2 Exposures

The main exposure variables were internalized and enacted stigma. Internalized stigma was defined as whether respondents reported feeling ashamed because of their HIV status [9]. Responding “yes” meant that the PLHIV had experienced internalized stigma. Enacted stigma, on the other hand, was operationalized as respondents’ self‐reported experience of discrimination attributable to their HIV‐positive status [10]. This construct captured realized stigma expressed through unfair treatment, exclusion, or negative reactions from others. Participants were asked whether they had ever experienced discriminatory actions because of their HIV status. Those who had experienced at least one of the discriminatory actions were classified as having experienced enacted stigma.

#### 2.2.3 Covariates

Informed by previous studies on HIV status disclosure and HIV stigma [18, 19, 20, 21], the following covariates were selected: age, level of education, media exposure, place of residence, wealth index, HIV transmission knowledge, marital status, and region of residence. For example, Berhe et al. [19] highlight the significance of HIV knowledge and counseling on status disclosure. Dessalegn et al. [20] and Adams et al. [21] also document how marital status can influence HIV disclosure. HIV transmission knowledge was measured using a composite binary indicator derived from three items assessing correct understanding of vertical transmission pathways (i.e., transmission during pregnancy, delivery, and breastfeeding). Each item had binary response options (yes/no). A respondent was classified as having adequate HIV transmission knowledge (1) only if all three items were answered correctly. Participants who answered one or more items incorrectly were categorized as having inadequate knowledge (0).

### 2.3 Statistical Analyses

The statistical analyses were completed in STATA version 14 (StataCorp, College Station, TX, USA). The ZDHS has a complex survey structure. And so, there was a need to account for this. Consequently, we applied the survey weights while accounting for the primary sampling unit. We then computed the weighted descriptive statistics to summarize the sample characteristics. Following that, bivariate associations between each explanatory variable and HIV disclosure were examined using survey‐weighted cross‐tabulations with row proportions. Multivariable logistic regression models were later fitted to estimate adjusted odds ratios (AORs) and 95% confidence intervals for factors associated with HIV disclosure. All covariates were entered simultaneously into the model. Robust standard errors were used to account for the complex survey design. The variance inflation factor was used to account for multicollinearity. In this case, the VIF value was less than 10, and thus, indicative of an absence of problematic multicollinearity. The Pseudo R‐square was reported to show the amount of variation explained by the regression model. Statistical significance was set at *p* < 0.05.

## 3 Results

### 3.1 Characteristics of the Sample

Among the 1037 PLWHIV in this study, 26.1% and 21.8% experienced internalized and enacted stigma, respectively (Table 1). Nearly two‐thirds had complete HIV transmission knowledge (62.7%). Most of the participants were aged 40–44 (22.8%), had secondary education (46.5%), were currently in union (56.6%), resided in urban areas (65.0%), resided in Lusaka (26.1%), and lacked regular media exposure (71.7%). Most participants were in the richer (29.3%) and richest (26.4%) quintiles.

**Table 1:** Sample weight distribution and prevalence of HIV status disclosure.

Characteristics	Weighted sample	Disclosed HIV status	*p*‐value
	*n* (%)	*n* (% [95% CI])	
**Overall**	1037 (100.0)	930 (89.7 [87.5–91.5])	
**Internalized stigma**			**0.004**
No	766 (73.9)	700 (91.4 [89.0–93.3])	
Yes	271 (26.1)	229 (84.6 [79.4–88.8])	
**Enacted stigma**			0.536
No	811 (78.2)	724 (89.4 [86.9–91.4])	
Yes	226 (21.8)	205 (90.7 [86.4–93.8])	
**HIV transmission knowledge**			**0.003**
Incomplete knowledge	387 (37.3)	333 (86.0 [81.9–89.2])	
Complete knowledge	650 (62.7)	597 (91.9 [89.5–93.7])	
Age group (years)			0.143
15–19	24 (2.3)	21 (88.0 [67.7–96.3])	
20–24	43 (4.2)	34 (79.0 [62.5–89.4])	
25–29	114 (11.0)	98 (86.0 [78.3–91.3])	
30–34	211 (20.3)	187 (88.6 [82.9–92.5])	
35–39	220 (21.2)	198 (90.2 [85.4–93.5])	
40–44	237 (22.8)	216 (91.1 [86.2–94.4])	
45–49	189 (18.2)	176 (93.3 [88.7–96.1])	
**Education**			0.292
No education	68 (6.6)	58 (85.8 [76.0–92.1])	
Primary	421 (40.6)	375 (89.1 [85.8–91.6])	
Secondary	483 (46.5)	434 (89.9 [86.4–92.7])	
Higher	66 (6.3)	63 (95.4 [88.0–98.3])	
**Marital status**			0.802
Never married	122 (11.8)	107 (88.1 [80.6–92.9])	
Currently in union	587 (56.6)	528 (90.1 [87.4–92.3])	
Previously in union	328 (31.7)	294 (89.5 [85.7–92.4])	
**Media exposure**			0.239
No	744 (71.7)	662 (88.9 [86.4–91.1])	
Yes	293 (28.3)	268 (91.5 [87.5–94.3])	
**Residence**			**< 0.001**
Urban	674 (65.0)	630 (93.4 [90.8–95.3])	
Rural	363 (35.0)	300 (82.7 [78.6–86.2])	
**Wealth index**			**0.003**
Poorest	111 (10.7)	96 (86.3 [79.4–91.2])	
Poorer	147 (14.2)	121 (82.4 [75.8–87.5])	
Middle	202 (19.5)	182 (90.1 [85.3–93.5])	
Richer	303 (29.3)	272 (89.8 [85.6–92.8])	
Richest	274 (26.4)	258 (94.4 [90.2–96.9])	
**Region**			**0.014**
Central	134 (12.9)	126 (94.2 [88.7–97.1])	
Copperbelt	156 (15.0)	136 (87.1 [79.1–92.3])	
Eastern	93 (8.9)	80 (86.8 [77.7–92.5])	
Luapula	55 (5.3)	47 (86.7 [77.3–92.6])	
Lusaka	271 (26.1)	253 (93.5 [88.9–96.2])	
Muchinga	24 (2.3)	22 (93.3 [79.1–98.1])	
Northern	27 (2.7)	22 (80.3 [65.1–89.9])	
North Western	37 (3.5)	33 (90.2 [78.9–95.8])	
Southern	139 (13.4)	126 (90.9 [84.7–94.7])	
Western	102 (9.8)	83 (81.5 [73.8–87.3])	

*Note:* Bold values indicate statistical significance.

### 3.2 Prevalence of HIV Among Women in Zambia

Overall, 89.7% (95% CI: 87.5–91.5) of women reported having disclosed their HIV status (Table 1). Disclosure prevalence was significantly higher among women who did not experience internalized stigma (91.4%) and among those with complete HIV transmission knowledge relative to those with incomplete knowledge (91.9%). However, the disclosure prevalence was higher among those who experienced enacted stigma (90.7%). Urban residents (93.4%), those aged 45–49 years (93.3%), those with higher education (94.5%), those with regular media exposure (91.5%), and women in the richest wealth index (94.4%) had a higher prevalence of HIV status disclosure. The highest disclosure was reported in Central (94.2%) and Lusaka (93.5%) provinces.

**Figure 1:**
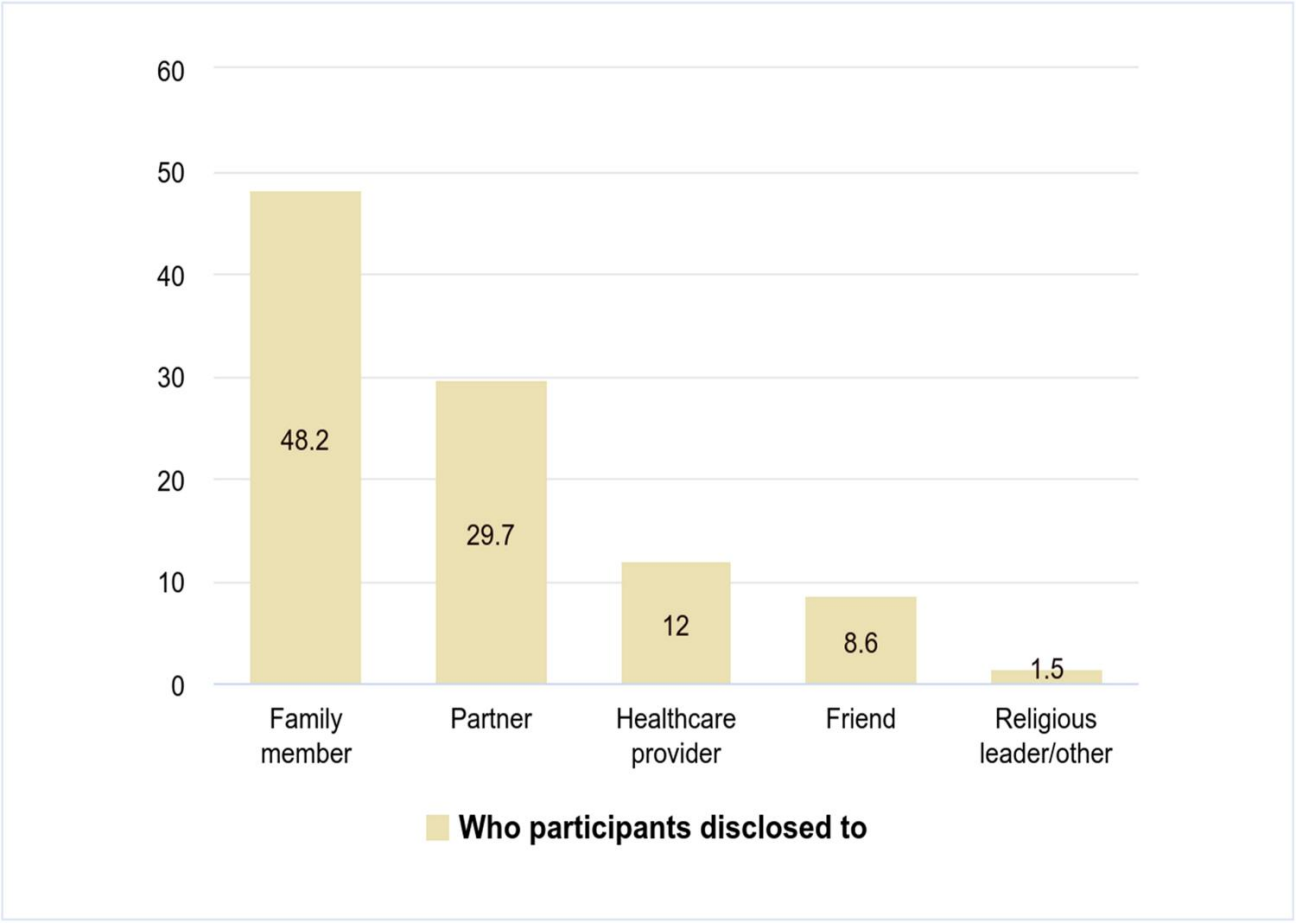
Who women living with HIV disclosed their status to.

The findings also revealed that women living with HIV mostly disclosed their status to family members (48.2%), followed by their intimate partners (29.7%) (Figure 1). Religious leaders were the least group (1.5%) to whom women living with HIV disclosed their status.

### 3.3 Association Between Internalized and Enacted Stigma and HIV Disclosure

Table 2 shows that in the unadjusted model, women who experienced internalized stigma had significantly lower odds of HIV disclosure [AOR = 0.53; 95% CI: 0.35–0.79]. However, enacted stigma showed no statistical significance. After adjusting for covariates, the pattern remained, with internalized stigma remaining significantly associated with lower odds of HIV disclosure [AOR = 0.52; 95% CI: 0.33–0.84]. Having complete knowledge about HIV transmission was associated with higher HIV status disclosure [AOR = 1.97; 95% CI: 1.27–3.05].

**Table 2:** Association between internalized and enacted stigma and HIV disclosure.

Characteristics	Model I	Model II
	COR [95%CI]	AOR [95%CI]
**Internalized stigma**		
No	Ref.	Ref.
Yes	**0.53 [0.35–0.79]** **	**0.52 [0.33–0.84]** **
**Enacted stigma**		
No	Ref.	Ref.
Yes	1.16 [0.72–1.89]	1.52 [0.89–2.62]
**HIV transmission knowledge**		
Incomplete knowledge	Ref.	Ref.
Complete knowledge	**1.96 [1.32–2.91]** **	**1.97 [1.27–3.05]** **
**Model fit statistics**		
Pseudo R2		0.1249
Prob > chi2		< 0.001

*Note:* VIF: 5.13; Model I: assessed the crude associations between stigma and HIV status disclosure. Model II: assessed the crude associations between stigma and HIV status disclosure, while adjusting for HIV transmission knowledge, age, education, place of residence, media exposure, marital status, wealth index, and region of residence. Bold values indicate statistical significance.

** *p* < 0.01.

## 4 Discussion

Historically, HIV in many sub‐Saharan African contexts has been associated with moral judgment, fear, and social exclusion, contributing to persistent stigma even in the era of widespread ART [2, 3, 4]. These social perceptions continue to shape disclosure decisions among women living with HIV [11, 12, 13]. This study sought to examine the association between internalized, enacted stigma and HIV status disclosure among women in Zambia. The findings indicate that HIV status disclosure is high in Zambia (89.7%). This high disclosure is similar to what has been reported in Tanzania (90%) [19], Nigeria (85.2%) [22], and Ghana (83.3%) [23]. The estimated prevalence of HIV status disclosure is higher than what has been reported in Ethiopia (66%) [18]. We argue that the high disclosure prevalence reflects years of large‐scale HIV awareness campaigns, routine provider‐initiated testing, and strong integration of counseling within ART services in Zambia [24].

The study also revealed that PLHIV disclosed their status more to family members than any other group. This observation is inconsistent with the findings of a systematic review [17] that concluded that PLHIV disclosed more to their partners than family members/relatives. However, it aligns with a cross‐sectional study conducted in Ghana [21] that found a 79% disclosure to family members. It is possible that the predominance of disclosure to family members rather than sexual partners suggests that disclosure decisions are driven primarily by anticipated emotional and material support rather than prevention‐oriented motives. In many Zambian households, family networks remain the primary source of care, financial assistance, and practical help with clinic attendance and medication adherence [25, 26].

Regarding the main aim of the study, we found that internalized and enacted stigma did not affect HIV status disclosure in the same way. Women who experienced internalized stigma were significantly less likely to disclose their HIV status. Our finding is corroborated by earlier cross‐sectional [27], cohort [28], and systematic review [29] studies that have found an inverse association between internalized stigma and HIV status disclosure. It must be noted that internalized stigma operates at the intrapersonal level, shaping self‐perception, shame, and anticipated rejection [28]. As such, women who internalize HIV‐related stigma tend to adopt negative societal beliefs about HIV as personal truths, leading to fear of judgment, diminished self‐worth, and concealment of status. Consequently, disclosure in this context becomes psychologically costly because it threatens identity, dignity, and social belonging. On the contrary, enacted stigma was associated with a 52% higher likelihood of disclosure, albeit not being statistically significant. This may be explained from the perspective of reverse association. By its nature, enacted stigma occurs outside the control of the PLHIV. This means that their status would need to be known to the outsiders before they can be subjected to unfair treatment, exclusion, or negative reactions from others. Also, the absence of statistical significance for enacted stigma may partly reflect insufficient statistical power to detect modest associations, heterogeneity in experiences of discrimination, or temporal sequencing issues inherent in cross‐sectional data.

The study also indicates that having a comprehensive knowledge of HIV transmission was associated with about 97% higher likelihood of disclosing one's HIV status. The observed association was expected as PLHIV with higher levels of knowledge about HIV transmission tend to have a reduced risk of HIV stigma [30]. This may encourage them to be confident to disclose their status to family members, friends, or other people. It underscores a need for Zambia's HIV services to strengthen structured educational components within counseling sessions.

## 5 Strengths and Limitations

Given that we relied on secondary data, we could not account for some other important confounders (e.g., perceived self‐efficacy, the cordiality between the PLHIV and partner, level of social support, etc.). Also, the cross‐sectional nature of the ZDHS means that causal inferences cannot be made. Therefore, the findings should be interpreted as mere statistical associations. HIV disclosure and stigma were all self‐reported, which makes it prone to recall bias. Another important limitation relates to the socioeconomic composition of the analytic sample. Women from the richer and richest wealth quintiles constituted a relatively larger proportion of the weighted sample compared to poorer women. This may affect the external validity of the findings because women from lower socioeconomic backgrounds often experience distinct structural vulnerabilities. We, however, worked to reduce this effect by applying survey weights to improve representativeness. The self‐reported measures of HIV disclosure and stigma may additionally be affected by social desirability bias, particularly because disclosure remains a sensitive issue. Furthermore, the cross‐sectional design limits temporal interpretation, making it impossible to determine whether stigma preceded disclosure behaviors or emerged as a consequence of disclosure experiences. Nonetheless, the dataset used was large enough to generalize to the larger population of women living with HIV in Zambia aged 15–49 years. It cannot be generalized to women older than 49 years.

## 6 Conclusion

Internalized and enacted stigma affect HIV status disclosure differently in Zambia. The Zambian Ministry of Health must intensify its efforts to empower PLHIV. The Zambian Ministry of Health must consider integrating cognitive‐behavioral counseling approaches, peer‐support interventions, stigma‐reduction campaigns, and community empowerment programmes within ART services. Such empowerment must address feelings of shame, self‐blame, and fear of rejection among PLHIV. Also, national HIV guidelines should mandate routine screening for internalized stigma within ART services and integrate structured counseling modules focused on self‐acceptance, resilience, and empowerment. This will help to minimize the incidence of internalized stigma and contribute to improving disclosure among PLHIV. Future studies can explore the reverse association between HIV disclosure and enacted stigma. Also, further qualitative studies are required to deeply explore the subjective experiences and perspectives that shape HIV status disclosure and enacted stigma.

## Author Contributions

**Joshua Okyere:** conceptualization, methodology, formal analysis, software, data curation, project administration, validation, writing – review and editing, writing – original draft. **Mutinta Monze:** conceptualization, methodology, project administration, writing – review and editing, writing – original draft, Validation.

## Funding

The authors have nothing to report.

## Ethics Statement

All methods are in accordance with the Declaration of Helsinki. As this is a secondary analysis, ethical approval was not applicable. However, the National Health Research and Training Institute (NHRTI) and the National Health Research Authority in Zambia granted ethical approval for the conduct of the ZDHS.

## Conflicts of Interest

The authors declare no conflicts of interest.

## Transparency Statement

The lead author (manuscript guarantor) affirms that this manuscript is an honest, accurate, and transparent account of the study being reported; that no important aspects of the study have been omitted; and that any discrepancies from the study as planned (and, if relevant, registered) have been explained.

## Data Availability

The data that support the findings of this study are openly available in the DHS Program at http://dhsprogram.com/data/available-datasets.cfm. The authors confirm that the data supporting the findings of this study are available in the Measure DHS repository: http://dhsprogram.com/data/available-datasets.cfm.

## References

[1] World Health Organization , HIV and AIDS: Key Facts. 2025, https://www.who.int/news-room/fact-sheets/detail/hiv-aids.

[2] Y. Lu , H. Li , R. Dong , et al., “Global and Regional Disease Burden of HIV/AIDS From 1990 to 2021 and Projections to 2030,” BMC Public Health 25, no. 1 (May 2025): 1928. 40413521 10.1186/s12889-025-23121-4 PMC12103032

[3] M. A. Liamba , N. Mukumbata , and S. K. Masenga , “Alarmingly High Recent Human Immunodeficiency Virus Infection Burden Among Newly Diagnosed Individuals in Mongu District, Zambia,” African Journal of Primary Health Care & Family Medicine 18, no. 1 (February 2026): 7. 10.4102/phcfm.v18i1.5202 PMC12969639 41773387

[4] L. B. Mulenga , J. Z. Hines , K. A. Stafford , et al., “Comparison of HIV Prevalence, Incidence, and Viral Load Suppression in Zambia Population‐Based HIV Impact Assessments From 2016 and 2021,” AIDS 38, no. 6 (May 2024): 895–905. 38227572 10.1097/QAD.0000000000003834 PMC11402832

[5] UNAIDS , Understanding Measures of Progress Towards the 95–95–95 HIV Testing, Treatment and Viral Suppression Targets, 2023, https://www.unaids.org/sites/default/files/media_asset/progress-towards-95-95-95_en.pdf.

[6] Ministry of Health, Zambia , Zambia Population‐Based HIV Impact Assessment (ZAMPHIA) 2021: Final Report (Lusaka, Ministry of Health, 2023), https://www.zamstats.gov.zm/wp-content/uploads/2024/01/ZAMPHIA-2021-Final-Report.pdf.

[7] T. Zurashvili , M. Pashalishvili , V. A. Earnshaw , et al., “Disclosing HIV Status to Sexual Partner: Findings From a People Living With Hiv Stigma Index 2.0 Study in the Country Georgia,” PLoS One 20, no. 10 (October 2025): e0331919. 41060901 10.1371/journal.pone.0331919 PMC12507297

[8] C. Bondarchuk , T. Lemon , V. Earnshaw , et al., “Disclosure Events and Psychosocial Well‐Being Among Young South African Adults Living With HIV,” International Journal of Behavioral Medicine 32, no. 1 (February 2025): 124–134. 38658438 10.1007/s12529-024-10291-5

[9] M. Pantelic , L. Sprague , and A. L. Stangl , “It's Not ‘All in Your Head’: Critical Knowledge Gaps on Internalized HIV Stigma and a Call for Integrating Social and Structural Conceptualizations,” BMC Infectious Diseases 19, no. 1 (March 2019): 210. 30832613 10.1186/s12879-019-3704-1 PMC6399894

[10] L. Ferguson , S. Gruskin , M. Bolshakova , et al., “Frameworks and Measures for HIV‐Related Internalized Stigma, Stigma and Discrimination in Healthcare and in Laws and Policies: A Systematic Review,” Journal of the International AIDS Society 25 (July 2022): e25915. 35818866 10.1002/jia2.25915 PMC9274352

[11] Joint United Nations Programme on HIV/AIDS , Global AIDS Strategy 2021–2026: End Inequalities (End AIDS, 2021).

[12] J. R. Hargreaves , T. Pliakas , G. Hoddinott , et al., “The Association Between HIV Stigma and HIV Incidence in the Context of Universal Testing and Treatment: Analysis of Data From the HPTN 071 (PopART) Trial in Zambia and South Africa,” Journal of the International AIDS Society 25 (July 2022): e25931. 35818869 10.1002/jia2.25931 PMC9274206

[13] J. R. Hargreaves , S. Krishnaratne , H. Mathema , et al., “Individual and Community‐Level Risk Factors for HIV Stigma in 21 Zambian and South African Communities: Analysis of Data From the HPTN071 (PopART) Study,” AIDS 32, no. 6 (March 2018): 783–793. 29369164 10.1097/QAD.0000000000001757 PMC5854764

[14] E. A. Felker‐Kantor , M. E. Wallace , A. S. Madkour , D. T. Duncan , K. Andrinopoulos , and K. Theall , “HIV Stigma, Mental Health, and Alcohol Use Disorders Among People Living With HIV/AIDS in New Orleans,” Journal of Urban Health 96, no. 6 (December 2019): 878–888. 31520231 10.1007/s11524-019-00390-0 PMC6904691

[15] C. Hong , A. M. Ochoa , B. D. Wilson , E. S. Wu , D. Thomas , and I. W. Holloway , “The Associations Between HIV Stigma and Mental Health Symptoms, Life Satisfaction, and Quality of Life Among Black Sexual Minority Men With HIV,” Quality of Life Research 32, no. 6 (June 2023): 1693–1702. 36648570 10.1007/s11136-023-03342-z PMC10953729

[16] Zambia Statistics Agency, Ministry of Health (MoH) [Zambia], and ICF , Zambia Demographic and Health Survey 2024 (Zambia Statistics Agency, MoH, and ICF, 2024), https://www.zamstats.gov.zm/wp-content/uploads/2025/11/Zambia-Demographic-and-Health-Survey-2024.pdf#page=37.68.

[17] J. Okyere and M. Monze , “State of Human Papilloma Virus Vaccine Uptake Among Girls (15–21 Years) in Zambia: Analysis of a 2024 National Survey,” Journal of Health, Population, and Nutrition 45, no. 1 (2026): 124. 41877260 10.1186/s41043-026-01301-1 PMC13137585

[18] D. J. Damian , D. Ngahatilwa , H. Fadhili , et al., “Factors Associated With HIV Status Disclosure to Partners and Its Outcomes Among HIV‐Positive Women Attending Care and Treatment Clinics at Kilimanjaro Region, Tanzania,” PLoS One 14, no. 3 (March 2019): e0211921. 30865633 10.1371/journal.pone.0211921 PMC6415788

[19] T. M. Berhe , L. Lemma , A. Alemayehu , D. Ajema , M. Glagn , and S. Dessu , “Research Article HIV‐Positive Status Disclosure and Associated Factors Among HIV‐Positive Adult Patients Attending Art Clinics at Public Health Facilities of Butajira Town, Southern Ethiopia,” AIDS Research and Treatment 2020, no. 1 (2020): 7165423. 33204528 10.1155/2020/7165423 PMC7665937

[20] N. G. Dessalegn , R. G. Hailemichael , A. Shewa‐Amare , et al., “HIV Disclosure: HIV‐Positive Status Disclosure to Sexual Partners Among Individuals Receiving HIV Care in Addis Ababa, Ethiopia,” PLoS One 14, no. 2 (February 2019): e0211967. 30768642 10.1371/journal.pone.0211967 PMC6415764

[21] A. Adam , A. Fusheini , M. A. Ayanore , et al., “HIV Stigma and Status Disclosure in Three Municipalities in Ghana,” Annals of Global Health 87, no. 1 (June 2021): 49. 34164262 10.5334/aogh.3120 PMC8212837

[22] A. O. Temitayo‐Oboh , O. O. Adedokun , and M. C. Asuzu , “Disclosure of HIV Status and Associated Factors Among People Living With HIV/AIDS (PLWHA) in Ibadan, Nigeria,” Annals of Health Research 10, no. 4 (December 2024): 332–342.

[23] A. Agbeko , R. Owusu , Y. Alhassan , T. Letsa , W. K. Axame , and D. Ogum‐Alangea , “HIV Status Disclosure and Quality of Life of People Living With HIV/AIDS in the Ho Municipality, Ghana,” Advances in Public Health 2022, no. 1 (2022): 6842957.

[24] A. B. Heri , F. L. Cavallaro , N. Ahmed , M. M. Musheke , and M. Matsui , “Changes over Time in HIV Testing and Counselling Uptake and Associated Factors Among Youth in Zambia: A Cross‐Sectional Analysis of Demographic and Health Surveys From 2007 to 2018,” BMC Public Health 21, no. 1 (March 2021): 456. 33676482 10.1186/s12889-021-10472-x PMC7937241

[25] M. Chanda Prudence , HIV Disclosure Patterns Among Sexually Active Adolescent Girls and Young Women Living With HIV in Urban Communities of Zambia (Stellenbosch University, 2021).

[26] R. Masa , M. Zimba , G. Zimba , G. Zulu , J. Zulu , and D. Operario , “The Association of Emotional Support, HIV Stigma, and Home Environment With Disclosure Efficacy and Perceived Disclosure Outcomes in Young People Living With HIV in Zambia: A Cross‐Sectional Study,” Journal of the Association of Nurses in AIDS Care 35, no. 1 (January 2024): 17–26. 10.1097/JNC.0000000000000442 PMC10842355 37994517

[27] C. Mugo , D. Seeh , B. Guthrie , et al., “Association of Experienced and Internalized Stigma With Self‐Disclosure of HIV Status by Youth Living With HIV,” AIDS and Behavior 25, no. 7 (July 2021): 2084–2093. 33389374 10.1007/s10461-020-03137-0 PMC8768004

[28] M. Davtyan , D. Kacanek , J. Lee , et al., “The Role of Internalised HIV Stigma in Disclosure of Maternal HIV Serostatus to Children Perinatally HIV‐Exposed but Uninfected: A Prospective Study in the United States,” Journal of the International AIDS Society 26 (October 2023): e26167. 37909234 10.1002/jia2.26167 PMC10618870

[29] K. Gabbidon , T. Chenneville , T. Peless , and S. Sheared‐Evans , “Self‐Disclosure of HIV Status Among Youth Living With HIV: A Global Systematic Review,” AIDS and Behavior 24, no. 1 (2020): 114–141. 30924065 10.1007/s10461-019-02478-9

[30] S. B. Letshwenyo‐Maruatona , M. Madisa , T. Boitshwarelo , et al., “Association Between HIV/AIDS Knowledge and Stigma Towards People Living With HIV/AIDS in Botswana,” African Journal of AIDS Research 18, no. 1 (January 2019): 58–64. 30880585 10.2989/16085906.2018.1552879

